# Tuning of dynamic solvation structures via click chemistry for PEO-based solid polymer electrolytes

**DOI:** 10.1038/s41598-025-16709-8

**Published:** 2025-10-02

**Authors:** Ruiyang Li, Xueying Yang, Qichen Chen, Boyang Huang, Siyu Zhao, Peng Zhang, Jie Lin, Jinbao Zhao

**Affiliations:** 1 College of Chemistry and Chemical Engineering, State-Province Joint Engineering Laboratory of Power Source Technology for New Energy Vehicle, State Key Laboratory of Physical Chemistry of Solid Surfaces, Engineering Research Center of Electrochemical Technology, Ministry of Education, Collaborative Innovation Center of Chemistry for Energy Materials, Xiamen University, Xiamen, 361005 China; 2 College of Energy, Xiamen University, Xiamen, 361102 China; 3 Anhui Provincial Key Laboratory of Advanced Catalysis and Energy Materials, Ultra High Molecular Weight Polyethylene Fiber Engineering Research Center of Anhui Province, Anqing Normal University, Anqing, 246133 China; 4 Department of Engineering Science, University of Oxford, Oxford, OX1 3PJ UK; 5 School of Mechanical and Aerospace Engineering, Queen’s University Belfast, Belfast, BT9 5AH UK

**Keywords:** Lithium-ion batteries, Solid polymer electrolyte, Poly(ethylene oxide), Click chemistry, Li^+^ transport, Batteries, Batteries

## Abstract

The Li^+^-transport mechanisms in both solid polymer electrolytes (SPEs) and liquid electrolytes (LEs) are fundamentally governed by solvation dynamics, requiring an optimal balance between continuous coordination and moderate binding strength. Poly(ethylene oxide) (PEO) is a classic SPE matrix that leverages its –CH_2_–CH_2_–O– (EO) segments to provide continuous oxygen coordination for Li^+^ transport via amorphous regions. While continuous EO segments facilitate the intra-chain Li^+^-transport, their strong multidentate solvation of Li^+^ through a chelate effect – each Li^+^ chelates with 4–6 ethylene oxide (EO) units – significantly hinders the inter-chain Li^+^ mobility. This effect creates rigid solvation cages that both immobilize Li^+^ and resist modification by alternative moieties (e.g. carbonate or nitrile groups), resulting in poor room-temperature ionic conductivity (*σ*) and low Li^+^ transference number (*t*_Li+_). To address these challenges, we developed a series of precise Li^+^-transport models (LTMs) through click chemistry, strategically combining acrylate-PEG and acrylonitrile to engineer balanced interactions between multidentate (EO) and monodentate (C = O, C ≡ N) coordination sites. This design achieved synergistic enhancement of both inter- and intra-chain transport pathways, demonstrated by significantly improved performance with *σ* = 6.40 × 10^− 5^ S/cm and *t*_Li+_ = 0.44 at 25 °C. This approach permits tailored control of dynamic solvation structures, offering new opportunities to enhance Li^+^ transport in PEO-based solid polymer electrolytes.

**Supplementary Information:**

The online version contains supplementary material available at 10.1038/s41598-025-16709-8.

## Introduction

Lithium-ion batteries (LIBs) have become the dominant electrochemical systems for electrified transportation and utility-scale energy storage, playing a pivotal role in sustainable energy transition toward global net-zero targets^1,2^. However, commercial LIBs with liquid electrolytes (LEs) and graphite anodes are approaching their theoretical limits of energy density at ~ 300 Wh/kg, struggling to meet the high-energy demand of electrified transportation, such as heavy-duty electric vehicles, ships, and aircrafts. Solid-state lithium-ion batteries (SSLBs) equipped with solid-state electrolytes (SSEs) and lithium metal (theoretical specific capacity ~ 3860 mAh/g) are regarded as one promising high-energy battery solution. SSEs exhibit inherent low flammability, good leak resistance and high mechanical rigidity to potentially suppress lithium dendrite growth and enhance battery safety. Among SSEs, solid polymer electrolytes (SPEs)^3–6^ stand out for their superior processability and interfacial compatibility, making them particularly suitable for seamless integration into existing battery manufacturing processes.

In many electrolytes, Li^+^ transport is fundamentally governed by the dynamics of solvated Li^+^ ions, as the majority of Li^+^ exists in coordinated states with solvent molecules^7–9^. This Li^+^-solvation is shared in both SPEs and common LEs, where Li^+^ is solvated with polar moieties, such as ether (–O–), carbonyl (C = O) and ester groups (–COO–) after dissolution and dissociation of lithium salts, leading to analogous ion transport mechanisms^10–12^. However, their transport behaviors diverge significantly due to distinct physicochemical properties arising from their molecular or chain structures. In LEs, Li^+^-transport follows a vehicular mechanism, in which small independent solvent molecules form a solvation sheath around Li^+^, and the entire complex migrates collectively under an electric field. In contrast, SPEs exhibit a structural mechanism dominated by polymer segmental motions, where Li^+^ hops between coordination sites through continuous coordination-discoordination processes along the polymer chains. Consequently, the ionic conductivity (*σ*) and Li^+^ transference number (***t***_Li+_) of SPEs are determined by the dynamic solvation structure of polymer matrices, particularly the ability of Li^+^-solvation and the segment mobility of polymer chains for continuous coordination-discoordination processes of Li^+^ at various sites.

Poly(ethylene oxide) (PEO) is a classic SPEs matrix due to its continuous –CH_2_–CH_2_–O– (EO) backbones^13–16^ which provides sufficient Li^+^ coordination sites and high segment mobility for Li^+^-transport with low glass transition temperature (*T*_g_), suitable electronegativity for Li^+^ coordination-discoordination, and capability of reorganizing dynamic solvation structure^9,17^. The continuous EO backbones provide sufficient Li^+^ coordination sites in polymer matrix and flexible segmental mobility for intrachain Li^+^-transport in structural mechanism. Therefore, the amorphous EO segments are flexible enough to form a dynamic solvation structure and transport Li^+^ through intra-chain along with polymeric Li^+^-solvation structure at the macromolecular level, directly affecting the *σ* of PEO. However, the intrinsic chain flexibility and continuous distribution of O atoms in EO segments promote the formation of a chelate-like Li^+^-solvation structure at the micromolecular level, characterized by tight (EO)_4~6_–Li^+^ interactions—a macromolecular analogue of the chelate effect observed in G4 ((EO)_5_–Li^+^ interactions)^7,9^. This chelate-like coordination gradually constructs a polymeric solvation cage (namely (EO)_n_–Li^+^) that hinders the discoordination of Li^+^ from the existing solvation structure and confines Li^+^ within a single chain, reducing the chain flexibility. Furthermore, the polymeric solvation cages deeply couples Li^+^-transport with the multi-scale structure of PEO from microscale short- and long-range chain structures to the macroscale crystallization^9^, eventually resulting in a poor overall Li^+^-transport behavior with low *σ* and ***t***_Li+_. Therefore, releasing Li^+^ from (EO)_n_–Li^+^ to promote its dynamic solvation structure is crucial for improving *σ* and ***t***_Li+_ of PEO.

In order to improve the *σ* and ***t***_Li+_ of PEO, plenty of strategies have been proposed, such as introduction of fillers (particles, fibers or polymers)^18,19^, development of cross-linked polymer backbones^20^ and novel design of molecular structure^11,12,21^. However, most of these strategies have primarily focused on a single structural scale modification (i.e., reducing crystallinity or designing molecule chain), while overlooking the fundamental role of Li^+^-solvation structure dynamics in Li^+^ transport. Although EO groups facilitate intrachain Li^+^ transport, their multidentate and continuous coordination sites hinder interchain Li^+^ transport. On the contrary, the ester (C = O) and nitrile (C ≡ N) groups provide monodentate and discontinuous coordinate sites whose classic SPE matrices are poly(ethylene carbonate) (PEC) and poly(acrylonitrile) (PAN), respectively^10,22,23^. Due to the lack of ether group, ester-based and nitrile-based SPEs have rigid segments that come with low *σ* but can promote interchain Li^+^ migration. Concurrently, the large dipole moments of ester and nitrile groups result in loose solvation structure and accelerate Li^+^-discoordination and the dynamic changes of the solvation structure. Mindemark’s group^24,25^ reported that changing coordinating sites from ether to ester could significantly increase ***t***_Li+_. The ***t***_Li+_ of poly(trimethylene carbonate) (PTMC) and poly(ε-caprolactone) (PCL) with ester groups are greater than 0.6, superior to that of PEO series (***t***_Li+_~0.2). The same improvement of ***t***_Li+_ was found in PAN-based polymer-in-salt electrolyte, where it could be as high as 0.47 as nitrile groups of PAN tended to form aggregated ionic clusters, restricting the movement of large anions while facilitating Li^+^ transport through a smaller monodentate coordination structure^26^. Hence, monodentate coordination sites provided by ester and nitrile groups allow to tune Li^+^-solvation structure without the chelate effect, leading to the further improvement of ***t***_Li+_. This finding suggests a major focus toward tuning the dynamic solvation structure at the molecular level.

To achieve this goal, it is necessary to quantify solvation structures formed by different functional groups in polymer chains. Click chemistry^27,28^ is a simple, efficient, and selective synthesis method which is suitable for quantitative introduction of functional groups to regulate solvation structure. Among these, thiol-ene click reaction is a particular effective method for forming C-S bonds through the radical polymerization between thiol and alkene. For the accurate and quantified design of novel SPEs^29–31^, pentaerythritol tetrakis(3-mercaptopropionate) (PETMP) with four thiol groups is typically employed to provide thiol groups, while acrylate-terminated polyethylene glycols derivatives (acrylate-PEGs) function as the alkene donor. This thiol-ene method enables precise control over the number of repeating EO units in the EO segment and the molar ratio of EO segments to coordination groups within a molecule. Thus, the thiol-ene reaction serves as a crucial approach for the precise and quantitative design of polymer structures, enabling the tuning of dynamic solvation structures and elucidating the physicochemical interplay between polymer architecture and Li^+^-transport at the molecular level.

**Fig. 1:**
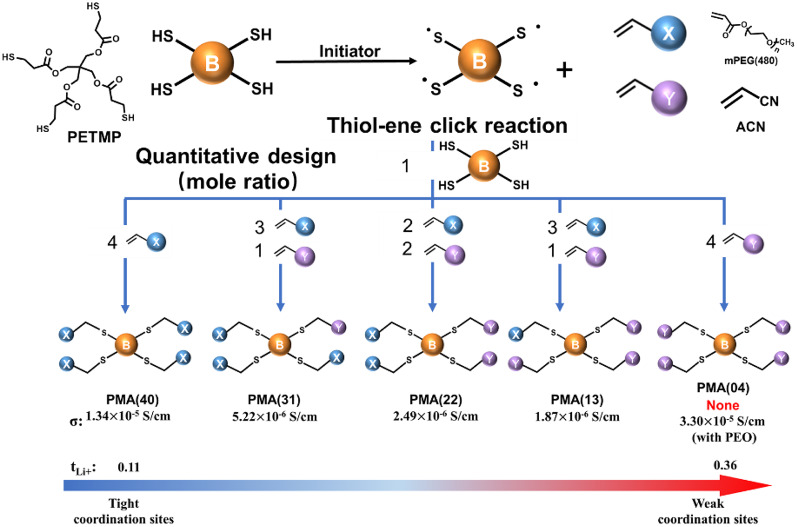
Quantization design of LTMs and the effect on *σ* and ***t***_**Li+**_.

In this work, a series of SPE matrices were synthesized via the thiol-ene reaction, serving as Li^+^-transport models (LTMs) for the study of solvation preferences and fine tuning of dynamic solvation between monodentate and multidentate coordination sites, as well as exploring their distinct effects on inter- and intra-chain Li^+^-transport. As shown in Fig. 1, this precise synthetic approach enables quantitative control over polymer architecture to fundamentally examine how tailored coordination environments influence Li^+^ transport mechanisms at a molecular level. We choose PETMP as a tetrafunctional thiol core, which is strategically modified through sequential thiol-ene reactions with acrylate-PEG (providing EO and C = O) and acrylonitrile (ACN, providing C ≡ N) to produce five LTM systems with different distributions of monodentate (C = O and C ≡ N) and multidentate (EO) coordination sites: PMA(40) (4 acrylate-PEGs), PMA(31) (3 acrylate-PEGs + 1 ACN), PMA(22) (2 acrylate-PEGs + 2 ACN), PMA(13) (1 acrylate-PEGs + 3 ACN), and PMA(04) (4 ACNs). Comprehensive FTIR demonstrate that while the multidentate EO segments form stable (EO)_4~6_–Li^+^ polymeric solvation cages at lower LiTFSI concentrations, the monodentate nitrile and ester groups become increasingly competitive in Li^+^ coordination as salt concentration increases. Most significantly, by progressively replacing multidentate EO sites with monodentate C ≡ N groups, we achieve substantial improvements in both ionic conductivity and Li^+^ transference number, where respective *σ* and ***t***_Li+_ increase to 3.33 × 10^− 5^ S/cm and 0.36 for the PMA(04)/PEO system. Solid-state NMR reveal that the monodentate-rich PMA(04) effectively modulates the typically rigid (EO)_4~6_–Li^+^ solvation structure of PEO, creating more dynamic coordination environments that facilitate Li^+^ transport while maintaining sufficient coordination strength for salt dissociation.

## Results and discussion

**Fig. 2:**
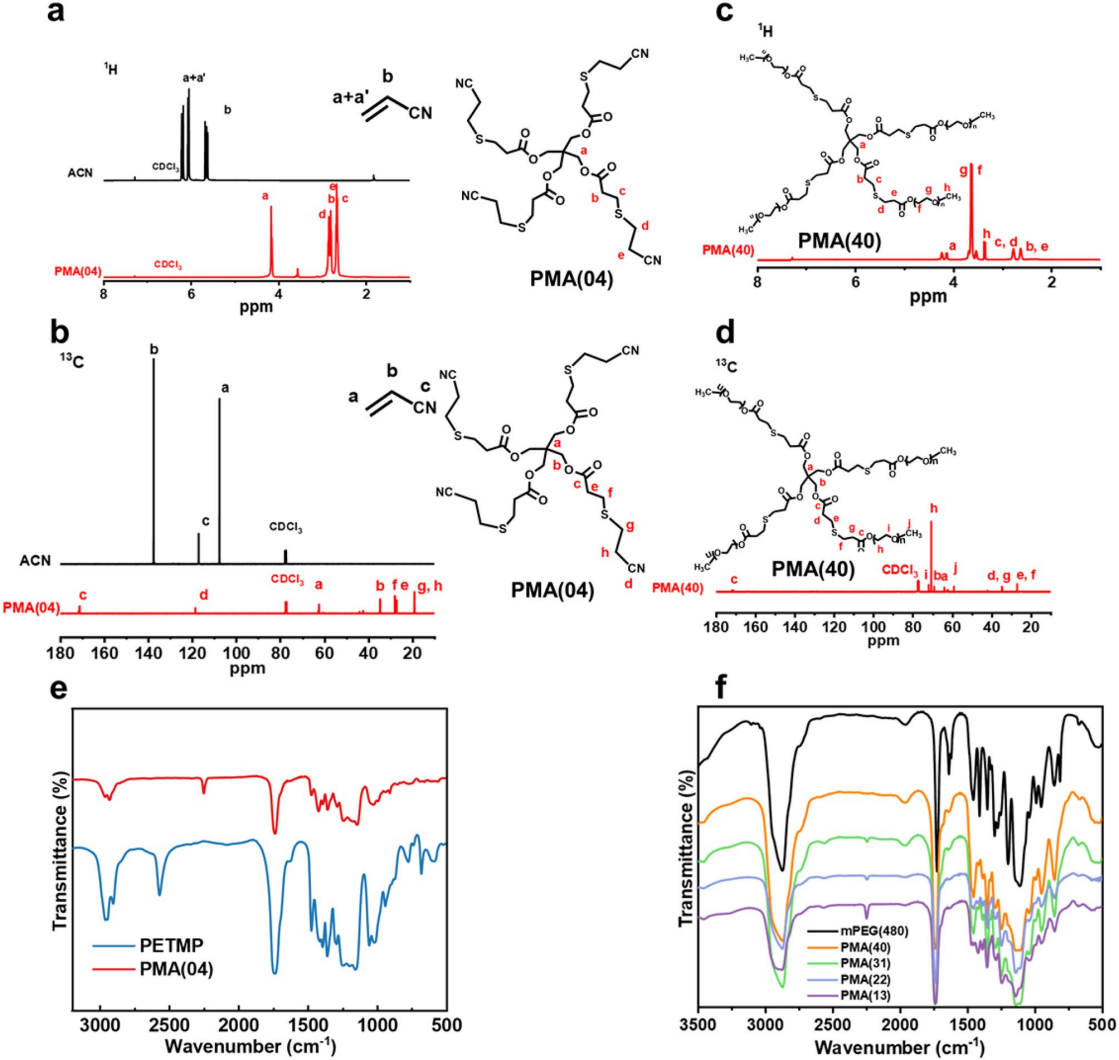
The molecule structure of LTMs. (a) ^1^H and (b) ^13^C NMR of ACN and PMA(04); (c) ^1^H and (d) ^13^C NMR of PMA(40). FTIR spectra of (e) PETMP, PMA(04) and (f) mPEG(480), PETMP, PMA(40), PMA(31), PMA(22) and PMA(13).

The synthesis route of LTMs is shown in Fig. S1. The ^1^H and ^13^C NMR of PMA(04) and PMA(40) are shown in Fig. 2a-d, respectively. Note the ^1^H NMR of ACN: *δ* = 6.2-6.0 (C***H***_***2***_ ***=*** CHCN), 5.7–5.6 (CH_2_ ***=*** C***H***CN); The ^13^C NMR of ACN: *δ* = 106.79 (***C***H_2_ ***=*** CHCN), 137.39 (CH_2_ ***= C***HCN), 117.10 (CH_2_ ***=*** CH***C***N). The ^1^H and ^13^C NMR of PMA(04) verified the disappearance of vinyl groups and the appearance of nitrile (C ≡ N) groups, and the ^1^H NMR and ^13^C NMR of PMA(40) verified the disappearance of vinyl groups and the appearance of EO groups. Furthermore, the FTIR shown in Fig. 2e and f also revealed the molecule structures of PMA(04) and PMA(40), respectively. The disappearance of thiol groups at approximately 2740 cm^− 1^ (which is consistent with the ^1^H NMR data of PETMP shown in Fig. S4), along with the appearance of ester (C = O), C ≡ N, and EO groups at around 1750, 2240, and 1100 cm^− 1^, respectively, could be confirmed in the FTIR spectra of PMA(04) and PMA(40). As shown in Table S3, the N/S mole ratios of PMA(04) and PMA(40) were measured by an elemental analyzer and consistent with their theoretical values, proving the quantitative relationship of each group. By verifying the molecule structures of LTMs using NMR and FTIR, it was confirmed that PMA(04) and PMA(40) were successfully synthesized via the thiol-ene click reaction, providing abundant coordination sites for tuning the dynamic solvation structure.

**Fig. 3:**
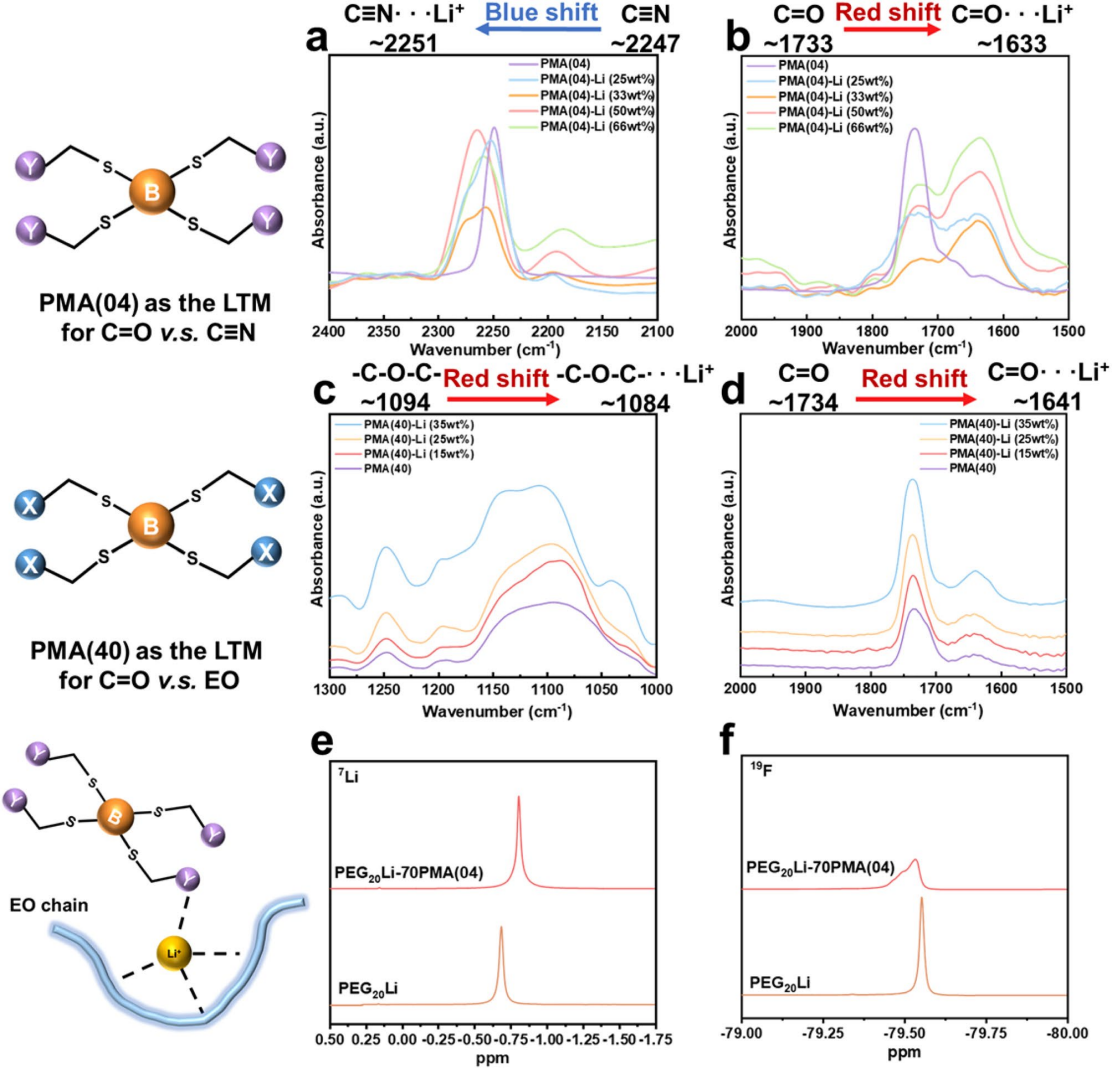
The Li^+^-solvation structure of LTMs. The FTIR of PMA(04) and PMA(04)-Li ranging (a) from 2400 to 2100 cm^-1^ and (b) from 2000 to 1500 cm^-1^; The FTIR of PMA(40) and PMA(40)-Li ranging (c) from 1300 to 1000 cm^-1^ and (d) from 2000 to 1500 cm^-1^. (e) ^7^Li and (f) ^19^F NMR spectra of PEG_20_Li and PEG_20_Li-70PMA(04).

To investigate Li^+^ coordination preferences in LTMs, FTIR spectra (Fig. 3a-d) and mole ratios of repeating units to LiTFSI (Table 1) were analyzed for PMA(04)-Li and PMA(40)-Li. Li^+^ coordination with polar moieties (C ≡ N, C = O, EO) induces distinct FTIR peak shifts. In PMA(04)-LiTFSI (PMA(04)-Li) system, pristine PMA(04) (see Fig. 3a and b) exhibits C ≡ N and C = O peaks at 2247 cm^–1^ and 1733 cm^–1^, respectively, which is consistent with the literature^10,32,33^. Upon LiTFSI addition, new peaks emerge at 2254–2264 cm^–1^ for C ≡ N···Li^+^ (Fig. 3a) and 1633 cm^− 1^ for C = O···Li^+^ (Fig. 3b). The C ≡ N···Li^+^ peak broadens due to two overlapping C ≡ N and C ≡ N···Li^+^ states (Δ*ν* < 20 cm⁻¹), while C = O···Li^+^ shows resolved splitting (Δ*ν* ≈ 100 cm^− 1^). Furthermore, the ability of Li^+^-solvation of C ≡ N and C = O were investigated under different weight ratios of LiTFSI. As shown in Fig. 3a, increasing the weight ratio of LiTFSI in PMA(04)-Li progressively shifts C ≡ N···Li^+^ peak from 2247 cm^−1^ (C ≡ N in PMA(04)) to 2251 cm^− 1^ (25 wt%), 2256 cm^− 1^ (33 wt%) and 2264 cm^− 1^ (50 wt%). Above 50 wt%, C ≡ N···Li^+^ peak reverses to 2258 cm^− 1^ (66 wt%). On the contrary, the C = O···Li^+^ peak is fixed at around 1633 cm^− 1^ with peak intensity growing monotonically at higher LiTFSI weight ratio. This result indicates that Li^+^ tends to coordinate with C = O rather than C ≡ N at high LiTFSI concentration.

For PMA(40)-LiTFSI (PMA(40)-Li) system, the comparison between C = O and EO moieties is shown in Fig. 3c and d. The C = O peak in FTIR shifts from 1734 (pristine PMA(40)) to 1641 cm^− 1^ (PMA(40)-Li). At low LiTFSI concentrations (15–25 wt%), weak C = O···Li^+^ signals suggest dominant EO coordination which shifts from 1094 cm^− 1^ (EO) to 1084 cm^− 1^ (EO···Li^+^) in Fig. 3c. At 35 wt% LiTFSI and above, the coordination capacity of EO saturates, leading to an intensified C = O···Li^+^ peak (1641 cm^− 1^). Compared with the C = O···Li^+^ peak in PMA(04)-Li at 1633 cm^− 1^, the peak in PMA(40)-Li (1641 cm^− 1^) exhibits a slight blue shift for 8 cm^− 1^, reflecting a tight coordination competition between EO and C = O that is not observed between C ≡ N and C = O in PMA(04) due to the weaker coordination ability of C ≡ N. The tight chelate-like (EO)_4~6_–Li^+^ solvation structure dominates at low LiTFSI concentration and will prevent Li^+^ from coordinating with other moieties, but cannot fully exclude C = O···Li^+^ coordination at higher LiTFSI loadings.

While rigid (EO)_4~6_–Li^+^ cages limit complete Li^+^ transfer to monodentate C = O and C ≡ N sites, their partial destabilization enables Li^+^ migration between intrachain (EO-bound) and interchain (C = O/C ≡ N-mediated) pathways. This interdependence, captured by the blue shift in FTIR spectra, creates a dynamic solvation equilibrium where EO’s multidentate binding coexists with monodentate coordination, balancing restricted and mobile Li^+^ transport. Therefore, constructing monodentate and multidentate sites and tuning their ratios emerge as a critical strategy for optimizing solvation dynamics in solid polymer electrolytes.

To investigate the Li^+^-solvation structure during coordination competition between PEO and PMA(04), the ^7^Li and ^19^F NMR spectra of PEG_20_Li-70PMA(04) (i.e., 70 wt% PMA(04)) were analyzed in Fig. 3e and f. In NMR, PEG (*M*_v_=1000 Da) is often used as a liquid-like PEO model due to their similar multiscale structure, ensuring comparable Li^+^-solvation environments. PEG_20_Li-70PMA(04) exhibited optimal electrochemical performance among other weight ratios of PMA(04). In ^7^Li NMR spectra (Fig. 3e), the chemical shift of PEG_20_Li at − 0.69 ppm moves upfield to − 0.81 ppm for PEG_20_Li-70PMA(04), indicating increased electron density around Li^+^ due to additional coordination ligands. This suggests that PMA(04) acts as a LTM and participates in the Li^+^-solvation structure, likely through multidentate (EO)_4~6_–Li^+^ interactions. This finding is further corroborated by the ^19^F spectra in Fig. 3f. The chemical shift moves slightly downfield from − 79.6 ppm (PEG_20_Li) to − 79.5 ppm (PEG_20_Li-70PMA(04)), indicating a decreasing amount of TFSI^−^ in the Li^+^-solvation shell. These results show that PMA(04) in PEO has a similar effect to PMA(40) on the Li-solvation structure of PEO. As shown in Fig. S3, the phenomenon of the ^7^Li spectrum shifting towards the downfield can be observed with the addition of PMA(04) and PMA(40). The incorporation of PMA(04) and PMA(40) to provide monodentate (C = O and C ≡ N) and multidentate (EO) coordination sites, tend to achieve the tuning of dynamic solvation structure which disrupts the rigid (EO)_4~6_–Li^+^ cages, promoting a more dynamic solvation environment that facilitates both inter- and intra-chain Li^+^ transport.

**Table 1:** The mole ratio of repeating units to LiTFSI in PMA(04)-Li and PMA(40)-Li.

EO vs. C ≡ N	Composition	*n* (C = O + C ≡ N)/ *n* (LiTFSI)
PMA(04)-Li	PMA(04)-Li (25 wt%)	20
	PMA(04)-Li (33 wt%)	12
	PMA(04)-Li (50 wt%)	7
	PMA(04)-Li (66 wt%)	3

EO vs. C = O	Composition	*n* (C = O + EO)/ *n* (LiTFSI)
PMA(40)-Li	PMA(40)-Li (15 wt%)	22
	PMA(40)-Li (25 wt%)	13
	PMA(40)-Li (35 wt%)	9

**Fig. 4:**
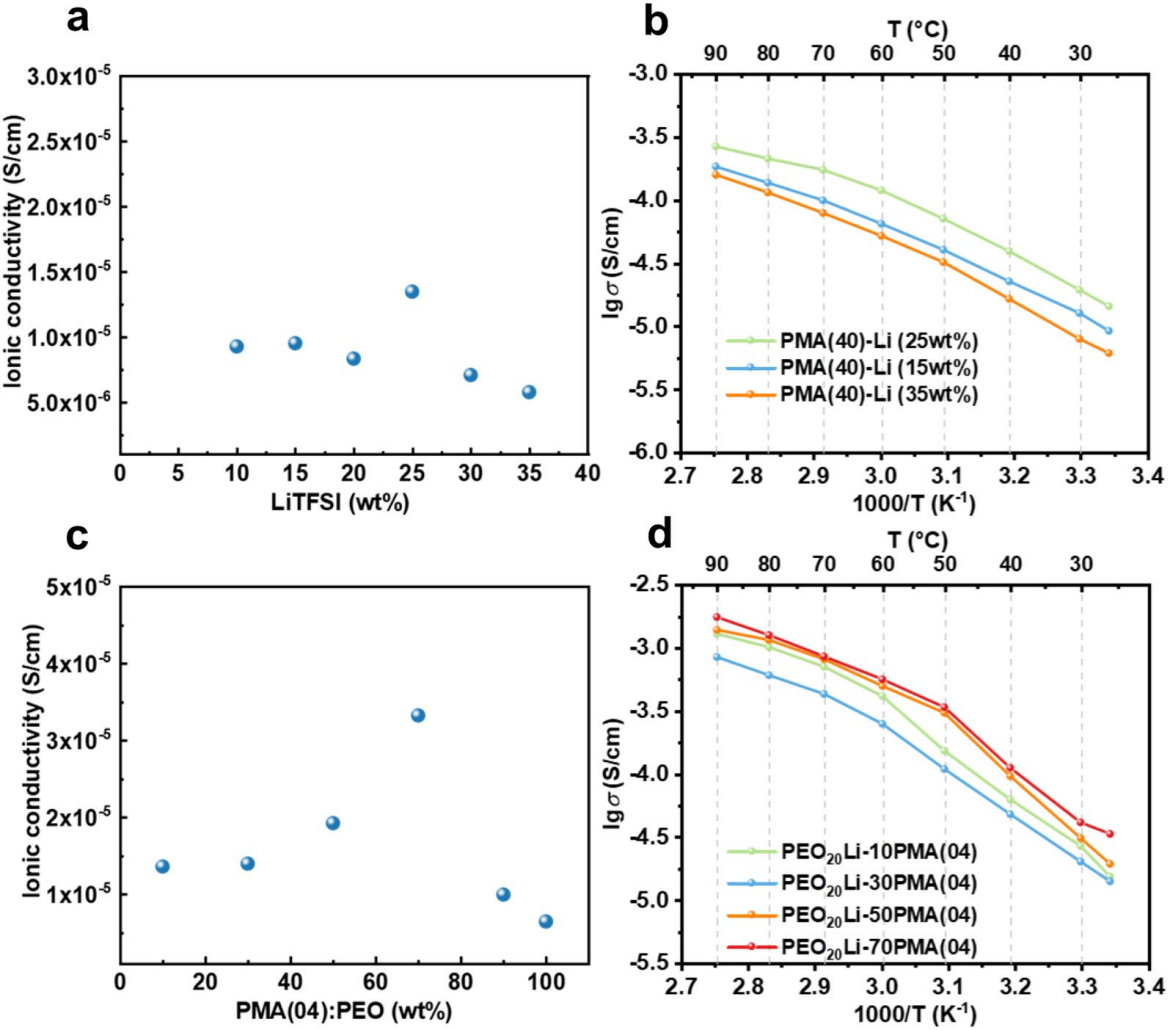
Electrochemical performances of LTMs. The *σ* of (a) PMA(40)-Li and (c) PEO_20_Li-*x*PMA(04) at 25 °C. Arrhenius plots of (b) PMA(40)-Li and (d) PEO_20_Li-*x*PMA(04).

The Li^+^-solvation structure is closely linked to Li^+^ transport in LTMs through structure-dependent mechanisms. Multidentate (EO)_4~6_ tend to form a cage-like coordination that promotes a continuous solvation environment favorable for intrachain Li^+^ transport. In contrast, monodentate C = O and C ≡ N groups lead to a discontinuous solvation environment, which are more conducive to interchain Li^+^ transport. Therefore, blending PEO with LTMs introduces continuous multidentate coordination sites and tuning the dynamic solvation environment, ultimately enhancing Li^+^ transport efficiency. The ionic conductivity *σ* and Arrhenius plots of PMA(40)-Li and PEO-PMA(04)-Li are provided in Fig. 4a-d. The *σ* values of PMA(40)-Li electrolytes with varying LiTFSI concentrations at 25 °C are 9.29 × 10^− 6^ (10 wt%), 9.54 × 10^− 6^ (15 wt%), 8.35 × 10^− 6^ (20 wt%), 1.35 × 10^− 5^ (25 wt%), 7.09 × 10^− 6^ (30 wt%) and 5.78 × 10^− 6^ S/cm (35 wt%), respectively (Fig. 4a). The maximum *σ* occurs at 25 wt% LiTFSI, suggesting an optimal balance between Li^+^ concentration and segmental mobility of EO chains for ion transport. Arrhenius plots (20–90 °C) reveals that PMA(40)-Li (25 wt%) consistently exhibits higher *σ* than those of 15 wt% and 30 wt% samples. The linear Arrhenius behavior further confirms that Li^+^ transport in PMA(40)-Li occurs primarily through amorphous EO segments, with minimal influence from crystalline domains. As for PMA(04), their abundant monodentate coordinate sites facilitate tuning of the dynamic solvation structure, hence PEO is blended with PMA(04) to obtain a suitable ability of Li^+^-transport. The *σ* values of PEO_20_Li-PMA(04) at 25 °C are 1.36 × 10^− 5^ (10 wt%), 1.40 × 10^− 5^ (30 wt%), 1.93 × 10^− 5^ (50 wt%), 3.33 × 10^− 5^ (70 wt%), 9.98 × 10^− 6^ (90 wt%) and 6.47 × 10^− 6^ S/cm (100 wt%), respectively (Fig. 4c). The maximum ionic conductivity (*σ* = 3.33 × 10^− 5^ S/cm) is achieved at 70 wt% PMA(04) content, which is attributed to the reduced crystallinity of PEO. The Arrhenius plots of PEO_20_Li-PMA(04) systems exhibit non-linear behaviors (Fig. 4d), suggesting that Li^+^ transport is impeded by the crystalline domains within the polymer matrix. Notably, the inflection point in the Arrhenius plots shifts to lower temperatures as the PMA(04) content increases from 10 to 70 wt% (Fig. S2), consistent with a progressive reduction in crystallinity (Table S2) that enhances Li^+^ mobility.

In both PMA(40)-Li (25wt%) and PEO_20_Li-70PMA(04), the highest *σ* is in the order of 10^− 5^ S/cm at 25 °C, revealing that Li^+^-transport is dominated by EO segments. However, the *t*_Li+_ of PEO_20_Li-70PMA(04) at 0.36 is higher than that of PMA(40)-Li (25wt%) (*t*_Li+_=0.11), reflecting their different molecule structures. In PMA(40)-Li, while the rich EO segments achieve suitable *σ*, they also introduce tight (EO)_4~6_–Li^+^ coordination cages, which restrict Li^+^ mobility between different coordination sites and suppress *t*_Li+_. In contrast, PMA(04) lacks EO segment but contains a large amount of monodentate C = O and C ≡ N moieties. These groups weaken Li^+^-polymer interactions, which promotes moderate coordination environments while not conducive to *σ* improvement, thereby enhancing *t*_Li+_. These results highlight a critical design principle that enhancing both *σ* and *t*_Li+_ requires balancing sufficient EO segments (for ion conductivity) with moderate coordination sites (for Li^+^ mobility). Specifically, the rigid (EO)_4~6_–Li^+^ solvation structure must be destabilized to facilitate interchain Li^+^ transport, while retaining enough EO content to sustain intrachain conduction pathways.

**Fig. 5:**
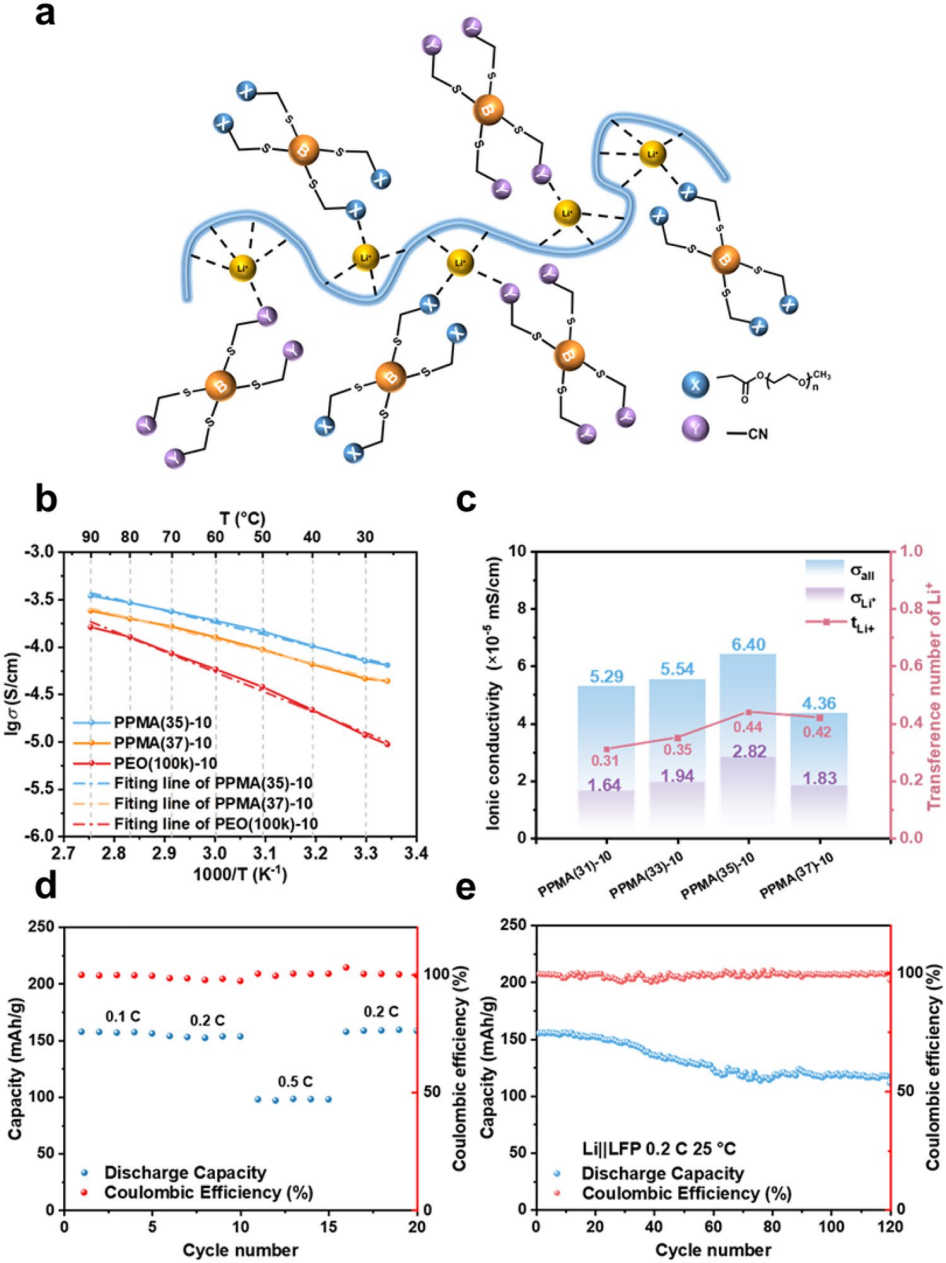
Electrochemical performances of SPEs improved by LTMs. (a) The dynamic solvation structure constructed by PEO(100k), PMA(40) and PMA(04). (b) Arrhenius plots of PPMA(35)-10, PPMA(37)-10 and PEO(100k)-10. (c) The *σ* and ***t***_Li+_ of PPMA(31)-10, PPMA(33)-10, PPMA(35)-10 and PPMA(37)-10 at 25 °C. The cycling performance of Li||LFP cells with PPMA(37)-10 (d) at different rates (0.1 C, 0.2 C and 0.5 C) and (e) at 0.2 C at 25 °C.

Figure 5a demonstrates that a moderate Li^+^-solvation structure constructed by PEO(100k), PMA(40) and PMA(04) with tunable dynamics is crucial for establishing efficient inter- and intra-chain Li^+^-transport channels in PEO-based SPEs, significantly enhancing their electrochemical performance. Electrochemical performances of SPEs improved by LTMs are shown in Fig. 5b–e, in which PMA(40) and PMA(04) were blended with PEO(100k) to construct optimized Li^+^-solvation structures through synergistic interactions among EO···Li^+^, C = O···Li^+^ and C ≡ N···Li^+^ coordination sites. While monodentate C = O···Li^+^ and C ≡ N···Li^+^ coordination sites in PMA(04) improve *t*_Li+_, they significantly reduce *σ* owing to restricted segmental mobility. The highest *σ* is 6.7 × 10^− 6^ S/cm at 25 °C when the weight ratio of PEO: PMA(04) is 1:0.3. To address this problem, PMA(40) with EO moieties was introduced to improve the Li^+^-solvation structure and Li^+^-transport. The *σ* of PPMA(35)-10, PPMA(37)-10 (see “Methods”) and PEO(100k)-10 were tested at different temperatures ranging from 25 to 90 °C (Fig. 5b) and the activation energy for ion transport (*E*_a_) could be derived by linear fitting of the Arrhenius equation (Table S1). The Arrhenius plots in Fig. 5b show that *σ* of PPMA(35)-10, PPMA(37)-10 and PEO(100k)-10 exhibit a continuous behavior with temperature increase owing to the amorphous structure of SPEs, which helps to clarify the tuning of dynamic solvation structures among intra-chain Li^+^-transport (long chain PEO) and inter-chain Li^+^-transport (LTMs). The *E*_a_ of PPMA(35)-10, PPMA(37)-10 and PEO(100k)-10 are 24.4, 25.2 and 41.1 kJ/mol, respectively. The lower energy barrier of PPMA(35) for ionic transport proves that the moderate Li^+^-solvation structure constructed by PMA(40) and PMA(04) facilitates the smooth movement of Li^+^-solvation structure and enhances Li^+^-transport (Fig. 5c). This tuning process initially increases both *σ* (from 5.29 × 10^− 5^ to 6.40 × 10^− 5^ S/cm) and *t*_Li+_ (0.31 to 0.44), followed by a decline due to excessive EO-bound solvation cage formation in PPMA(37). Ultimately, PPMA(35)-10 achieves the best *σ*_Li+_ through its balanced solvation structure, demonstrating the importance of moderate coordination environments for practical battery applications.

The Li||LFP cells were assembled with PPMA(35)-10 and evaluated at various C-rates (0.1–0.5 C) and 25 °C (Fig. 5d and e). The cells delivered stable discharge capacities of 156.0 mAh/g (0.1 C) and 152.4 mAh/g (0.2 C), demonstrating effective Li^+^ transport under moderate current densities. At 0.5 C, the capacity decreased to 97.7 mAh/g, attributed to the limited *σ*_Li+_ (2.82 × 10^− 5^ S/cm) being insufficient to sustain high-rate cycling. A long-term cycling test was performed at 0.2 C. The discharge specific capacity was 155.3 mAh/g at 1st cycle and retained 107.1 mAh/g at 120th cycle (67.0% retention). Notably, an obvious capacity fade occurred at ~ 40th cycle, which was caused by interfacial instability between C ≡ N groups in PMA(04) and Li metal, despite FEC pre-treatment of the lithium anode. Nevertheless, PPMA(35)-10 enabled relatively stable cycling at 25 °C, outperforming conventional PEO-based systems. These results confirm that PPMA(35)-10 successfully establishes a moderate Li^+^-solvation structure through balanced interactions between EO, C = O, and C ≡ N coordination sites. This optimized solvation environment facilitates efficient Li^+^-transport by simultaneously improving *σ* and *t*_Li+_, while mitigating the trade-offs typically observed in single-component SPEs.

## Conclusion

The dynamic Li^+^-solvation is crucial to the Li^+^-transport mechanisms of both SPEs and LEs, despite their different physical states. Effective Li^+^-transport, intrinsically driven by the dynamic movement of the Li^+^-solvation structure, requires a balanced solvation that combines continuous coordination with moderate binding strength to enable optimal ion mobility and enhanced Li^+^ conductivity ***σ***_Li+_. In this study, we combined PEO(100k) with LTMs to engineer such a structure through precisely tuned interactions between multidentate EO···Li^+^ and monodentate C = O···Li^+^/C ≡ N···Li^+^ coordination sites. The long-chain EO segments in PEO(100k) established continuous intrachain transport pathways, while the strategically incorporated PMA(40) and PMA(04) LTMs modified the polymeric solvation cages through their diverse coordination environments (EO, C = O and C ≡ N), accelerating interchain transport via optimized local electrostatic potentials and reduced steric effects. This synergistic design achieved a moderate Li^+^-solvation structure that simultaneously supported both inter- and intra-range Li^+^-transport mechanisms, yielding exceptional respective *σ* and *t*_Li+_ of 6.40 × 10^− 5^ S/cm and 0.44 at 25 °C. Unlike LEs where Li^+^-solvation occurs primarily at a single molecular scale, SPEs exhibit complex multiscale interactions from polymolecular to macromolecular where coordination behaviors of different moieties depend not only on their electrostatic potentials but also on their molecular weight, steric constraints, and aggregation states. The LTMs synthesized via click chemistry address these requirements by providing many precisely controlled coordination sites with tailored molecular weights, effectively destabilizing rigid polymeric solvation cages in long-chain EO moieties while maintaining sufficient coordination strength. This work demonstrates how dynamic tuning of EO, C = O, and C ≡ N interactions in LTMs can create optimized solvation structures that bridge long-range (intrachain) and short-range (interchain) transport pathways, offering a universal strategy for designing high-performance PEO-based SPEs.

## Methods

### Synthesis of Li^+^-transport models of SPEs via thiol-ene click reaction

The thiol–ene click reaction is an effective tool for modulating the polymer structure. The reaction is frequently promoted by a base with the abstraction of a H^+^ from a thiol added through the alkene C = C bond, forming a new C − S bond. The synthesis route is mentioned in Fig. S1. Pentaerythritol tetrakis(2-mercaptoacetate) (PETMP, Shanghai Aladdin Co., Ltd.) acts as thiol donor, Poly(oxy-1,2-ethanediyl) (mPEG, average Mn ~ 480, Sigma–Aldrich Co., Ltd.) and Acrylonitrile (AN, Shanghai Aladdin Co., Ltd.) act as alkene donor, and triethylamine (TEA, Sinopharm Group Co., Ltd.) as initiator. Five LTMs are designed with different distribution of moieties by different feed molar ratio. The LTMs obtained according to different feed molar ratio of PETMP: mPEG: AN = 1:4:0, 1:3:1, 1:2:2, 1:1:3, 1:0:4 are named as PMA(40), PMA(31), PMA(22), PMA(13) and PMA(04), respectively. The synthesis system is displayed in N’N dimethylformamide (DMF, Sinopharm Group Co., Ltd.) solvate with argon protected at room temperature. Firstly, moderate PETMP and TEA were added into 20 ml DMF stirring for 12 h to create enough thiolate anion. Secondly, mPEG and AN were dissolved in 30 ml DMF in a specific proportion respectively and added successively under argon protection for 12-hour thiol-ene click reaction. After that, in order to remove the residual monomer and DMF, the solution was drooped into cold diethyl ether and n-hexane for three times to precipitate. Eventually, the products via the same procedure were drying in a vacuum oven at 80 °C for 24 h to obtain LTMs for further discussion of Li^+^-transport in the molecular level.

### Preparation of PEO-based SPE and LTMs membranes

PEO (anhydrous, Aladdin) with *M*_v_ = 100,000 Da, lithium bis(trifluoromethanesulphonyl) imide (LiTFSI) (Alfa Aesar Co., China) and LTMs were dissolved together in the anhydrous acetonitrile (ACN, Aladdin) to obtain a homogeneous solution with 10 wt% solid mass is obtained after stirring intensely 12 h. After that, the solution was dropped into cellulose membrane (NKK TF4030) and then dried under 80 °C vacuum oven for 12 h. In this way, LTMs membranes blended with LiTFSI named as PMA(40)-Li(*x*) (*x* is the weight ratio of LiTFSI). As for PMA(04) without ionic conductivity, PMA(04) is blended with PEO and LiTFSI (O/Li^+^ = 20) named as PEO_20_Li-*a*PMA(04) (*a* refers to weight ratio of PMA(04)). As for, the PPMA-based SPE membranes, denoted as PPMA(cd)-n, were fabricated by dissolving predetermined ratios of PEO(*M*_v_ = 100,000 Da), LiTFSI and LTMs (PMA(04) and PMA(40)) in anhydrous acetonitrile, in which cd: Mass ratio of PMA(04) to PMA(40), n: EO/Li^+^ molar ratio. For instance, the membrane PPMA(35)-10 was prepared by mixing 1:0.3:0.5 mass ratio of PEO(*M*_v_ = 100,000 Da): PMA(04):PMA(40), EO/Li^+^ = 10 in 15 ml anhydrous acetonitrile. The homogeneous solution was cast onto a cellulose membrane substrate and dried under vacuum at 60 °C for 24 h to obtain freestanding SPE films.

### Assembly and measurements of batteries

In this study, all battery tests were performed using coin cells, i.e., CR2016 for lithium metal batteries and CR2032 for Li symmetric batteries. In lithium metal batteries, a lithium anode, a SPE membrane, and a LiFePO_4_ (LFP) cathode were sandwiched together. The symmetric cells were assembled by replacing the LFP cathode and lithium anode with stainless steel (SS) disks. The cathode was prepared by mixing LFP, 1,1-difluoroethylene homopolymer (PVDF), acetylene black, and 1,4-butanedinitrile (SN) into a solid solution (with 5 mol% LiTFSI in SN) with a mass ratio of 7:1:1:1, setting the mass loading of the LFP at around 1.3 mg/cm^2^. The cells were preheated under 60 °C for 1 h to ensure uniform Li^+^-transport between the electrodes and electrolyte. The electrochemical measurements were performed on a Neware multichannel battery testing system, cycling between 2.5 and 3.8 V.

### Characterization

^7^Li nuclear magnetic resonance (NMR) experiments were conducted on Bruker 500 MHz at room temperature, among which, PEG (*M*_v_ = 1000 Da, Aladdin) was used as the model of PEO due to its liquid state at room temperature after the addition of LiTFSI, named as PEG_20_Li. PEG_20_Li-30PMA(04) contained the weight ratio of PEG: PMA(04) = 1:0.3, and PEG_20_Li-30PMA(04)-30PMA(40) contained the weight ratio of PEG: PMA(04):PMA(04) = 1:0.3:0.3 for the study of dynamic solvation structure. The reference consisted of 0.1 M LiClO_4_ dissolved in D_2_O in a sealed capillary tube. The capillary tube was coaxially inserted into the NMR tube filled with the electrolyte sample. Differential scanning calorimetry (DSC) measurements were conducted on a Model STA 449 instrument (NETZSCH Machinery and Instruments Co., Ltd.) to investigate the enthalpy change of SPEs with a heating rate of 10 K/min.^7^Li solid-state wide cavity nuclear magnetic resonance (ssNMR) spectra were obtained from Bruker AVANCE NEO 600 MHz at room temperature. To investigate the interactions between different species in SPEs, the attenuated total reflection infrared (ATR-FTIR) spectroscopy was tested with a Nicolet IS5 spectrometer in the range of 600–4000 cm^− 1^. The N/S mole ratio of LTMs was evaluated on the elemental analyzer (Flash Smart CHNS/O, Thermo Fisher Scientific).

The ionic conductivity of PEO-based SPEs (membrane) was measured by electrochemical impedance spectroscopy (EIS) on an electrochemical Solartron workstation, and the frequency range was set at 0.1–10^5^ Hz with an amplitude voltage of 10 mV. Finally, the following formula is used to calculated ionic conductivity of SPEs:

$$\sigma = d/\left( {R_\text{b} \times A} \right)$$

where *σ* refers to the ionic conductivity of the electrolyte membrane, *d* refers to the thickness of the electrolyte membrane, *R*_b_ is the resistance of the electrolyte membrane and *A* is the area of the stainless steel (SS).

By employing the potentiostatic polarization method on an Autolab electrochemical workstation (Metrohm, Switzerland) with a Li/SPE/Li simulating cell, the lithium-ion transference number (*t*_Li+_) was calculated using the following equation:

$$t_{{\text{Li}^{ + } }} ~ = \frac{{I_{\text{SS}} \left( {\Delta V~ - ~I_{0} R_{0} } \right)}}{{I_{0} \left( {\Delta V~ - ~I_{\text{SS}} R_{\text{SS}} } \right)}}$$

where Δ*V* is 20 mV, *I*_0_ and *I*_SS_ are the initial and final current, and *R*_0_ and *R*_SS_ are the initial and final interfacial resistance.

## Supplementary Information

Below is the link to the electronic supplementary material.

Supplementary Material 1

## Data Availability

Data have been provided in full within the article and its supplementary information.
