# Supplementary material for: Tuning of dynamic solvation structures via click chemistry for PEO-based solid polymer electrolytes
Source: Sci Rep. 2025 Oct 2;15:34332. doi: 10.1038/s41598-025-16709-8 (PMC12491566; doi:10.1038/s41598-025-16709-8)
Supplement: Supplementary file 1 — Supplementary Material 1 [file 41598_2025_16709_MOESM1_ESM.docx]

**Supporting Information**

**Tuning of Dynamic Solvation Structures via Click Chemistry for PEO-Based Solid Polymer Electrolytes**

Ruiyang Li^a^, Xueying Yang^b^, Qichen Chen^a^, Boyang Huang^c^, Siyu Zhao^d^, Peng Zhang^b,*^, Jie Lin^e,*^, Jinbao Zhao^a,b,*^

^a^College of Chemistry and Chemical Engineering, State-Province Joint Engineering Laboratory of Power Source Technology for New Energy Vehicle, State Key Laboratory of Physical Chemistry of Solid Surfaces, Engineering Research Center of Electrochemical Technology, Ministry of Education, Collaborative Innovation Center of Chemistry for Energy Materials, Xiamen University, Xiamen, 361005, China

^b^College of Energy, Xiamen University, Xiamen 361102, China

^c^Anhui Provincial Key Laboratory of Advanced Catalysis and Energy Materials, Ultra High Molecular Weight Polyethylene Fiber Engineering Research Center of Anhui Province, Anqing Normal University, Anqing 246133, China

^d^Department of Engineering Science, University of Oxford, Oxford OX1 3PJ, United Kingdom

^e^School of Mechanical and Aerospace Engineering, Queen’s University Belfast, Belfast BT9 5AH, United Kingdom

*Corresponding authors.

E-mail addresses: jbzhao@xmu.edu.cn (J. Zhao), j.lin@qub.ac.uk (J. Lin), pengzhang@xmu.edu.cn (P. Zhang)


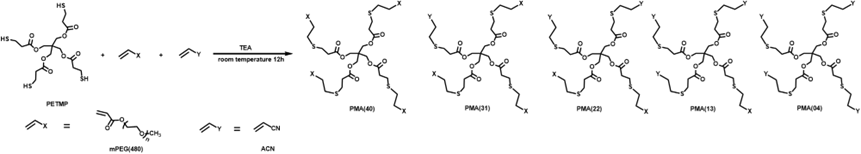


Fig. S1. The synthetic route of LTMs.

**Fig. S2.** The DSC of PEO_20_Li-10PMA(04), PEO_20_Li-30PMA(04), PEO_20_Li-50PMA(04) and PEO_20_Li-70PMA(04).


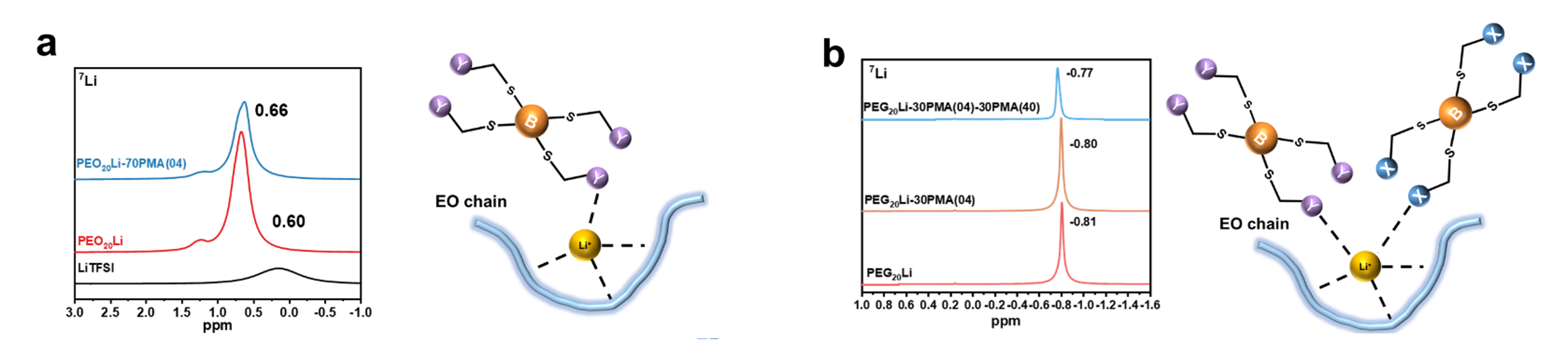


**Fig. S3** (a)The ^7^Li solid-state NMR (ssNMR) spectra of PEO_20_Li, PEO_20_Li-70PMA(04) and LiTFSI; (b) ^7^Li NMR spectra of PEG_20_Li, PEG_20_Li-30PMA(04) and PEG_20_Li-30PMA(04)-30PMA(40).

**
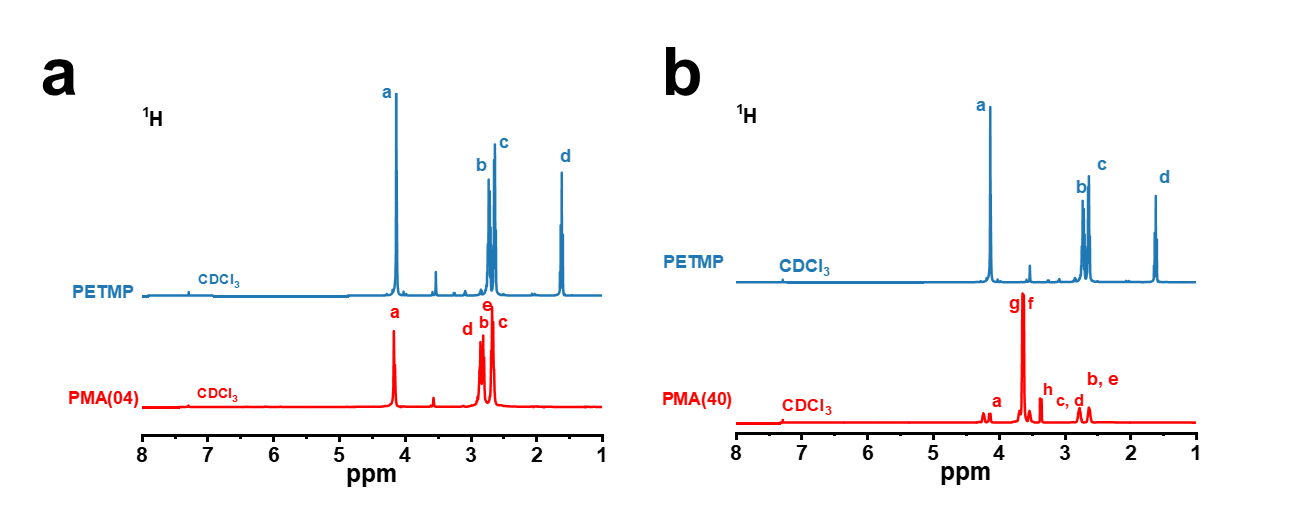
**

**Fig. S4** (a) ^1^H NMR spectra of PMA(04) and PETMP; (b) ^1^H NMR spectra of PMA(40) and PETMP.

Table S1 Activation energy (*E*_a_) and *R*^2^ of PPMA(35)-10, PPMA(37)-10 and PEO(100k)-10.

| Parameter | PPMA(35)-10 | PPMA(37)-10 | PEO(100k)-10 |
| --- | --- | --- | --- |
| *E*_a_ (kJ/mol) | 24.4 | 25.2 | 41.1 |
| *R^2^* | 0.994 | 0.995 | 0.995 |

Table S2 The crystallinity of PEO_20_Li-10PMA(04), PEO_20_Li-30PMA(04), PEO_20_Li-50PMA(04) and PEO_20_Li-70PMA(04).

| Composition | Crystallinity (%) |
| --- | --- |
| \| PEO-10PAH-20 \| \| --- \| \| PEO-30PAH-20 \| \| PEO-50PAH-20 \| \| PEO-70PAH-20 \| | 62.6  55.1  42.0  36.3 |

Table S3 The elemental analysis result of LTMs.

| Composition | N (wt%) | C (wt%) | H (wt%) | S (wt%) | N/S (mole ratio) |
| --- | --- | --- | --- | --- | --- |
| PMA(04) | 8.03 | 48.96 | 5.81 | 16.01 | 1.14 |
| PMA(13) | 3.94 | 49.98 | 6.99 | 11.63 | 0.77 |
| PMA(22) | 1.77 | 49.59 | 7.38 | 8.16 | 0.50 |
| PMA(31) | 1.09 | 50.76 | 8.10 | 9.54 | 0.26 |
| PMA(40) | 0.00 | 51.75 | 8.61 | 5.95 | 0.00 |
